# Prevalence and Associated Risk Factors of Intestinal Parasitic Infections: A Population-Based Study in Phra Lap Sub-District, Mueang Khon Kaen District, Khon Kaen Province, Northeastern Thailand

**DOI:** 10.3390/tropicalmed8010022

**Published:** 2022-12-27

**Authors:** Sirintip Boonjaraspinyo, Thidarut Boonmars, Nuttapon Ekobol, Atchara Artchayasawat, Pranee Sriraj, Ratchadawan Aukkanimart, Benjamabhorn Pumhirunroj, Panupan Sripan, Jiraporn Songsri, Amornrat Juasook, Nadchanan Wonkchalee

**Affiliations:** 1 Department of Community Medicine, Family Medicine and Occupational Medicine, Faculty of Medicine, Khon Kaen University, Khon Kaen 40002, Thailand; 2 Department of Parasitology, Faculty of Medicine, Khon Kaen University, Khon Kaen 40002, Thailand; 3 Department of Traditional Medicine, Faculty of Natural Resources, Rajamangala University of Technology ISAN Sakon Nakhon Campus, Sakon Nakhon 47160, Thailand; 4 Program in Animal Science, Faculty of Agricultural Technology, Sakon Nakhon Rajabhat University, Sakon Nakhon 47000, Thailand; 5 College of Allied Health Science, Suan Sunandha Rajabhat University, Samut Songkhram 75000, Thailand; 6 Faculty of Science and Technology, Phranakorn Rajabhat University, Bangkok 10220, Thailand; 7 Faculty of Veterinary Medicine, Mahasarakham University, Maha Sarakham 44150, Thailand; 8 Faculty of Nursing, Khon Kaen University, Khon Kaen 40002, Thailand

**Keywords:** intestinal parasite infection, prevalence, consuming raw food, *Opisthorchis viverrini*, anti-parasitic drug, Thailand

## Abstract

Intestinal parasitic infections are still a crucial problem among communities in Northeast Thailand. Misuse of antiparasitic drugs and unhealthy food behaviors are known. This study aimed to explore the prevalence, behavioral health factors, and motivation for self-treatment of anti-parasitic drugs in this area. A community-based cross-sectional study was conducted in Phra Lap sub-district, Mu Khon Kaen district, Khon Kaen province, Northeast Thailand, in 2016. A total of 419 participants were recruited to complete a self-administered questionnaire and stool examination. Binary logistic regression was used to assess the association between the risk factor and parasitic infection. Forty-two participants (10%; 95%CI 7.5–13.3) were positive for at least one parasite species. In this community, the most detected intestinal parasite was *Opisthorchis viverrini* (5.3%), followed by *Strongyloides stercoralis* (3.1%). A total of 67.5% of the participants had the experience of anti-parasitic drug treatment within previous 1 year, and “Often eat raw food” was the most common reason for the use of anti-parasitic drugs. On multivariate analysis, parasitic infections were significantly associated with male gender (ORadj. 2.42; 95%CI 1.00–5.85), age ≥ 60 years (ORadj. 7.55; 95%CI 1.60–35.76), and often consuming raw food of at least one type (ORadj. 2.37; 95%CI 1.03–5.44). Given these findings, correction of the dietary habit of eating raw fish/meat, which is the most important measure, and limitation of the use of anthelmintic treatment for individuals with stools positive for ova as well as emphasis on sanitary toilets will be implemented for the prevention and control of parasitic infection in endemic communities.

## 1. Introduction

Intestinal parasitic infection in the community is a neglected tropical disease, which is an important problem in developing countries. The global prevalence of soil-transmitted nematodes and Platyhelminthes remains high, about 30–807 million and 40–207 million, respectively [1]. The national survey of helminthiasis in Thailand showed that *Opisthorchis* (*O.*) *viverrini*, hookworm, and *Strongyloides* (*S.*) *stercoralis* remain important intestinal parasitic infections, particularly in the northeastern regions [2]. Infection with these parasites results in asymptomatic infection, but it is a chronic infection and interaction with the host immune response might contribute to human carcinogenesis and death if unrecognized. The fish-borne trematodes *O. viverrini* is classified by the International Agency for Research on Cancer (IARC) as a group 1 carcinogenic to humans for cholangiocarcinoma (CCA) [3]. Intestinal nematode *S. stercoralis* has a trend of causing lifelong infection and might develop into severe strongyloidiasis (hyperinfection syndrome or disseminated disease) in an immunocompromised host with a high mortality rate [4,5,6].

The prevention and control of intestinal parasitic infections in Thailand are focused on helminth infection. The Thai Department of Disease Control adopts active control in high-risk areas (parasite prevalence > 10%) by screening and treatment [7]. The mass drug administration for soil-transmitted helminth control program was recommended for all individuals in high-risk populations, i.e., wherein the prevalence was at least 20% without individual diagnosis [8,9]. Annual administration was implemented in Thai endemic areas (prevalence > 20%), especially in the school-age population [10]. A single 400 mg dose of albendazole was recommended for mass drug administration in combination with improved sanitation, water supply, and health education. The national decade strategy for opisthorchiasis was launched from 2016 to 2025 by the Ministry of Public Health. This 10-year-long strategy aims to eliminate the burden of liver fluke and bile duct cancer [11,12]. This policy introduced the early screening of liver fluke for risk populations aged more than 15 years old. Praziquantel treatment is strictly used in the “test-and-treat strategy”. Mass drug administration of praziquantel is recommended by the WHO [13,14], once a year if the liver fluke prevalence exceeds 20%. Mass treatment with 40 mg/kg for a single dose shows an effective cure rate of 95.9% with fewer side effects [15,16]. There is no recommended self-treatment with praziquantel, and this drug is rarely available at local drug stores.

Although there is a policy to control intestinal parasitic infections and the community is developing rapidly, local people still have a culture similar to the past times. They have a food culture that includes the consumption of improperly cooked fish or meat. The people believe that eating raw food and then taking anti-parasitic medicine will prevent parasitic infections. Self-treatment with over-the-counter anti-parasitic drugs from local stores is a common practice for people in these communities. Frequent use of praziquantel was thought to contribute to a risk of CCA in terms of repetitively inducing bile duct inflammatory reactions to dead worms [17]. The number of praziquantel treatments may increase the risk of CCA [18,19]. Antiparasitic drug treatment without a food behavior change will maintain the infection due to repeated exposures or infection events. Therefore, the local culture and inappropriate treatment are leading to the spread of parasites. There are many studies about the prevalence and risk factors of single parasitic infections, especially *O. viverrini* [20,21,22], but the intestinal parasites in communities are diverse, including protozoans and helminths. Little is known about the reason leading to the self-treatment of anti-parasitic drugs in the community and the association between self- treatment and having a parasitic infection.

Therefore, this study aimed to explore the prevalence of intestinal parasitic infection (helminth and protozoan), associated health behavioral factors, and the motivations for self-treatment of anti-parasitic drugs. These results will contribute to healthcare workers’ efforts to control and decrease the prevalence of parasite infection in communities.

## 2. Materials and Methods

### 2.1. Study Design and Setting

**Figure 1:**
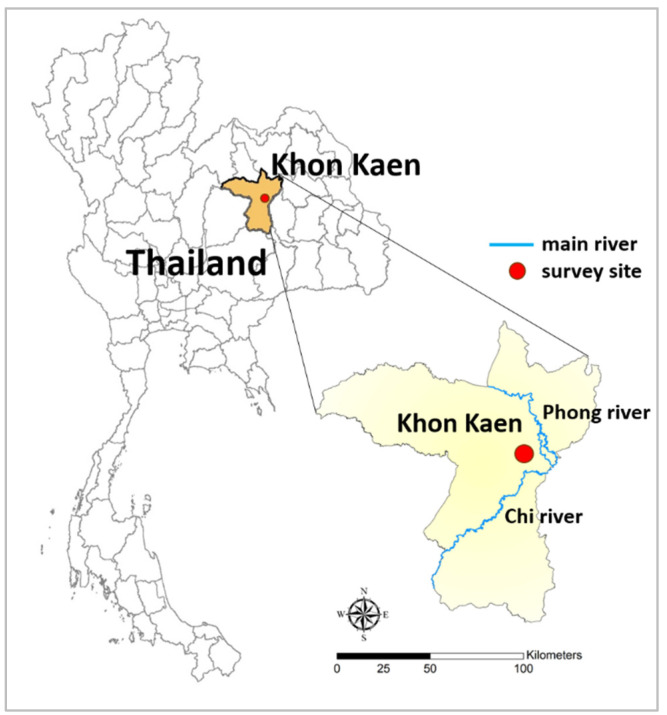
Location of the study area at Phra Lap sub-district, Mueang Khon Kaen district, Khon Kaen Province, northeastern Thailand.

A community-based cross-sectional survey was conducted between January and June 2016. The study was conducted in the Phra Lap sub-district, Mueang Khon Kaen district, Khon Kaen province, northeastern Thailand. The study area was semi-rural communities and was located along the Chi River (Figure 1). The average temperature was 27.8 °C and the annual rainfall was 1500.8 mm [23]. The total population in this area was 22,187.

### 2.2. Study Population and Sample

Participants more than 20 years of age were eligible for this study. Participants who were pregnant or had received anti-parasitic treatment for the last 1 month were excluded. The sample size for the estimated prevalence was calculated based on the finite population proportion [24] Formula (1) below:

n = [NZ^2^_1−α/2_ p(1 − p)]/[d^2^(N − 1) + Z^2^_1−α/2_ p(1 − p)] (1)

N: population size (N = 22,187)

Z^2^_1−α/2_: standard normal distribution with confidence interval of 95% (Z = 1.96)

p: the assumed proportion was 37.2% reported in a previous study [25] (p = 0.372)

d^2^: acceptable difference 5% (d = 0.05)

The sample size was 354. The final sample size was 443 after adjusting for a 20% expected loss of subjects. All participants were informed about the aims, procedures, potential risks, and benefits. Written informed consent was obtained from each participant. All protocols were approved by the Human Ethics Committee of Khon Kaen University, Khon Kaen, Thailand (ethical clearance no. HE581228). The participants were randomly selected in this study. Finally, a total of 419 participants returned stool specimens and questionnaires.

### 2.3. Parasitological Survey

Participants were instructed on the procedure for stool specimen collection and asked to provide a fresh morning stool in plastic containers. A self-administered questionnaire was used to collect socio-demographic data, data on self-treatment of anti-parasitic drugs, and health behavioral factors associated with parasitic infections. Illiterate participants were interviewed personally. The questionnaire was checked for validity by three independent parasitological experts and tested for reliability using Cronbach’s alpha coefficient with a reliability value of 0.892. The questionnaire and sample containers were marked with the participant identification number. All stool samples were stored in an ice box and processed on the same day using formalin-ether concentration methods [26,27] at the Parasitology Laboratory, Faculty of Medicine, Khon Kaen University. The fecal samples were weighed for 2 g and mixed with 10 mL of normal saline. The mixed sample was transferred to a plastic funnel with 2 layers of gauze and filtered to a new 15 mL test tube.The fecal solution was centrifuged at 1500 rpm for 2 min, the supernatant was discarded, 10 mL of normal saline was added, and the solution was centrifuged again for 2 to 3 rounds until the supernatant was a clearer color. Seven milliliters of 10% formalin was added and shaken. The formalin–fecal solution was stood for 5 min, then 3 mL of ethyl acetate was added and the solution was shaken vigorously. The solution was centrifuged again at 1500 rpm for 2 min. A wooden stick was used to de-plug the debris. The ethyl acetate, debris, and formalin were discarded. The processed specimen was sampled for two slides and examined by two parasitologists using a light microscope. Lugol’s iodine was used as the staining solution to detect intestinal protozoa and helminth from the specimen. A sample was considered positive if at least one parasite was detected in one slide. The participants who were infected with a parasite were treated with antiparasitic drugs and educated about the prevention of parasitic re-infection.

### 2.4. Statistical Analysis

The prevalence of intestinal parasites, reason for antiparasitic drug consumption, and frequency of raw food consumption among participants were reported by descriptive statistics. The subjects were divided into two groups: (1) intestinal parasitic infection and (2) non-parasitic infection according to the stool examination. Pearson’s chi-squared test was used to determine whether there was a statistically significant difference between these two groups with health behavioral factors. The Mantel–Haenzsel test for trend was used to assess whether there was a difference in the trend of the proportions in the two groups with ordered categories [28,29]. Binary logistic regression was used to assess the association between independent variables and parasitic infection. The variables based on the results of the univariable analyses and scientifically relevant factors were included in the multivariable analysis [30]. The statistically significant factors were considered as p-values less than 0.05 and reported with a 95% confidence interval. All statistical analyses were performed with SPSS Statistics 28.0 (IBM Corp., Armonk, NY, USA) and STATA10.0 software (Stata Corporation, College Station, TX, USA).

## 3. Results

### 3.1. Socio-Demographic Characteristics

A total of 419 people in communities participated in this study (230 females and 186 males). The 40–59-year-old age group was best represented (48.5%). The participants had largely completed primary school (71.2%), and many were working as agriculturists (37.8%). Nearly half of the people in the community (46.1%) had comorbidity. The most common comorbidities found were hypertension (21.0%), followed by diabetes mellitus (13.8%), hepatitis (3.8%), and gastritis (3.3%). Nineteen percent of participants were smokers or ex-smokers, and 32.7% either drank alcohol or were ex-drinkers (Table 1).

### 3.2. Prevalence of Intestinal Parasitic Infection

Of the 419 stool samples, 42 specimens (10%; 95%CI 7.5–13.3) were positive for at least one parasite species. The most common intestinal parasite in this community was *Opisthorchis viverrini* (5.3%), followed by *Strongyloides stercoralis* (3.1%), *Echinostome* spp., hookworm, *Ascaris lumbricoides*, and minute intestinal fluke were found in one case (0.2%) of each. The intestinal protozoa found in this study were *Blastocystis hominis, Entamoeba histolytica, E. coli*, and *Giardia lamblia* (Table 2).

### 3.3. Reason for Self-Treatment of Anti-Parasitic Drugs

**Figure 2:**
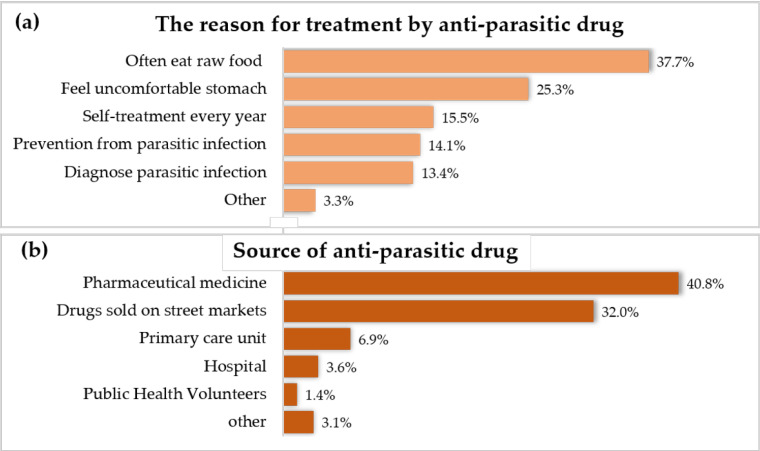
(**a**) Percentages of the reasons for treatment with an anti-parasitic drug; (**b**) source of anti-parasitic drugs. Note: participants could choose more than 1 answer.

Half of the participants (50.7%) had experienced parasitic infection screening, and from this group, 42% had a diagnosis of parasitic disease. A total of 81% of the participants in this community had the experience of anti-parasitic drug treatment. The most common reason for treatment with an anti-parasitic drug was “Often eat raw food” (37.7%), followed by “Feel uncomfortable stomach” (25.3%), “Self-treatment every year” (15.5%), and prevention from parasitic infection (14.1%). About 67.5% of the participants were treated with anti-parasitic drugs at least one time a year, and anti-parasitic drugs were commonly bought from pharmaceutical medicine stores (40.8%) and drugs sold on street markets (32.0%). More details are shown in Figure 2.

### 3.4. Sources of Food-Borne Parasitic Infections

**Figure 3:**
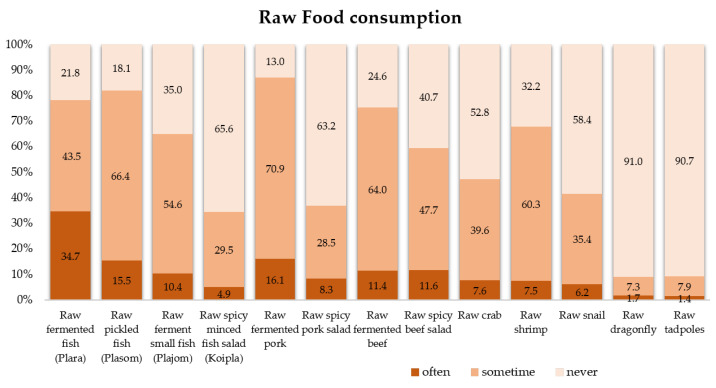
Percentage of raw food consumption among participants.

**Figure 4:**
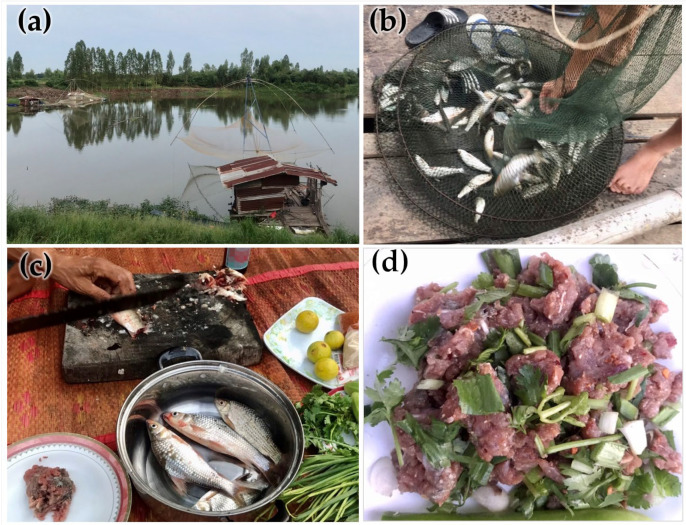
(**a**,**b**) The community located along the river where people fished for a living; (**c**,**d**) the traditional raw or improperly cooked fish consumption in this area.

The sources of food-borne parasitic infections in this community are shown in Figure 3. More than 30% of the participants in this area often consumed raw fermented fish followed by raw fermented pork (16.1%) and beef (11.4%). Fewer than 10% of the participants often consumed uncooked crab, shrimp, snail, dragonfly, and tadpoles. The traditional raw fish consumption and improperly cooked fish consumption in this area are shown in Figure 4c,d.

Table 3 summarizes the associations of all parasites and risk factors observed in univariable and multivariable regression analyses. The univariable analysis indicated that gender (OR_crude_ 3.08; 95%CI 1.55–6.12), smoking (OR_crude_ 4.36; 95%CI 2.05–9.30), alcohol consumption (OR_crude_ 3.05; 95%CI 1.51–6.16), and raw food consumption (OR_crude_ 2.65; 95%CI 1.23–5.73) were significantly associated with parasitic infection.

The multivariable analysis result was adjusted for age groups, gender, smoking, alcohol consumption, and raw food consumption. The significantly increased risks were observed in male participants for all parasitic infections (OR_adj_ 2.42, 95%CI 1.00–5.85) compared to females. Those older than 60 years of age showed a higher prevalence than the younger age group, and the difference was statistically significant (OR_adj_ 7.55; 95%CI 1.60–35.76). The odds of parasitic infection were 2.37-times more likely (OR_adj_ 2.37; 95%CI 1.03–5.44) for participants who often consumed raw food of at least one type compared to those who never or sometimes consumed raw food.

## 4. Discussion

One in ten of the participants in this community were infected with a parasite. The most common parasite was *O. viverrini*, a group 1 carcinogenic to humans for CCA [3], followed by *S. stercolaris*. The prevalence in this area was lower than reported in 2013 [25] and similar to several studies reporting a prevalence (6.2–16.1%) in different settings in Thailand [22,31,32,33]. The prevalence of protozoan infection (0.8%) was lower than helminth infection, similar to previous studies [34,35,36]. These previous studies reported that the prevalence of protozoan infection was commonly *Blastocystis hominis* (2.19–4.0%) [34,36] or giardiasis (0.6–1.28%) [35,36], which both had a higher prevalence than this study. However, protozoan infection was indicated to occur through the feacal–oral transmission route in food or water and poor sanitation in this area, which correspond to the result that participants infrequently used a toilet for defecation (37.4%). The decrease in parasitic infection prevalence in this area might result from the screening programs, treatment of infected participants, and health education from the active control program by government campaigning and research projects including self-treatment with anthelmintics.

Among the participants, more than two-thirds of participants use parasitic drugs at least once a year to avoid parasitic infection. The most common reported reason to use drug treatment was “Often eat raw food”. This result showed that the villagers knew raw food consumption could result in infection with a parasite. Similar to the study by Chaisiri et al., it was shown that the villagers are aware of the risks of raw food consumption [34]. In our study, the participants usually bought the drug from a pharmacy, but some villagers (~30%) bought the medicine from a street market. Moreover, the participants administered self-treatment without knowing the species of parasitic infection. The common anti-parasitic drugs in local stores are albendazole or mebendazole, both single-dose treatments. This regimen is not effective against opisthorchiasis or strongyloidiasis which require praziquantel or ivermectin as standard treatments [37,38]. Therefore, the medicine might not be specific to the parasite with which they are infected, resulting in incomplete treatment from an ineffective drug type and suboptimal dose. This may lead to continued *O. viverrini* infection and an increased risk of cholangiocarcinoma, especially in the northeastern part of Thailand, which is the endemic area of *O. viverrini* [39,40,41,42]. These results correspond to previous reports of inappropriate self-treatment of *O.viverrini* infection [43].

The factors associated with parasitic infections that were found in this study after analysis with confounder adjustment showed that being older than 60 years of age was associated with a higher chance of parasitic infection (by 7.55-fold) than younger age groups. This is consistent with the findings of previous studies, where the parasite infection rate increases with age [20,32]. Aging is a potential risk factor for parasitic infection. Protective immunity of the skin and gastrointestinal tract were weakened according to increased age [44,45,46]. Being an elderly person accompanied by comorbidity enhances the chance of being immunocompromised and thus susceptible to parasitic infection [47,48]. The culture of food preparation and food sharing is an important social factor for foodborne parasites in elder people. Misinformation about safe cooking and sharing of raw fish promote the risk of liver fluke infection in senior adults [49,50]. Additionally, males have a 2.4-fold increased risk of parasitic infection compared to females. This risk factor was also identified in previous studies [20,32,51,52]. This might be because males work outside of the home, especially in this area, where they mostly work as agriculturists and are likely to be in contact with the soil and not wearing shoes. The study by Wang et al. reported that males consume raw fish spicy salad 1.7 times compared to females because male participants believe that raw meat consumption provides power and strength. This finding highlights the misattribution of raw fish ingestion to masculinity [50]. Moreover, males working on farms might not properly cook their food and male fishers usually share raw fish dishes together with alcohol drinking [49], which can increase the risk of infection. Alcohol drinking and smoking were associated with parasitic infection in univariable analyses. Alcohol drinking and smoking increase the risk of *O. viverrini* infection [53,54]. Both first-hand and second-hand cigarette smoking promote the pathogenic immune response and/or weaken defensive immunity. This effect not only limits the airways but results in systemic pathology and lower systemic/mucosal protective immunity against parasitic infection [55]. Chronic alcohol consumption suppresses the protective immunity to parasitic infection. Alcohol stimulates the hypothalamus–pituitary–adrenal cortex axis to increase the production of cortisol. High levels of endogenous cortisol induce the polarization to Th1 dominant and induce Th2 cell apoptosis [56]. Moreover, alcohol can enhance the excystation of *O. viverrini* metacercaria [57]. The most important factor that is associated with an increased risk of parasite infection was often consuming raw food (at least one type, especially the consumption of raw fish or raw fermented fish). This area is located along the Chi River, where many people catch fish (cyprinid fish) for a living; there are many people who still had the behavior of consuming improperly cooked fish (Figure 4). This could account for the prevalence of *O. viverrini* infection in this area. Moreover, in this study, participants who often consumed raw food had a 2.37-fold higher rate of infection with parasites, similar to previous studies [21,31,33]. Additionally, the result of this study did not find an association between parasitic infection and the number of times a parasitic drug was used in 1 year. This finding indicated that self-treatment is not effective unless a behavioral change in raw food consumption and a proper regimen for parasitic control in endemic areas were taken up.

The findings of this study suggested that the risk factors for parasitic infection were (1) male gender, (2) greater than 60 years of age, and (3) frequent consumption of raw food (of at least one type of food). These risk factors might be valuable in a pre-screening for villagers screening themselves and village health volunteers for pre-screening the people before stool examination in communities. After stool examination, those who are found to be infected with parasites must be informed and treated to control parasitic diseases in the endemic area. The villagers need to be made aware that self-treatment does not prevent intestinal parasitic infections and in the case of opisthorchiasis may carry an additional risk of cholangiocarcinoma, thus emphasizing the importance of adequate cooking of fish and meat products.

A limitation of this study is that it used a cross-sectional study for risk factor analysis, which might affect the causal relationships between the risk pattern and parasitic infection.

## 5. Conclusions

The current study suggested that intestinal parasitic infection is still a problem in this area. This research highlights that the risk factors of parasitic infection were (1) male gender, (2) being 60 years of age or older, and (3) often consuming raw food. The screening programs will be implemented to prevent and control intestinal parasite infections in endemic communities. Furthermore, health education is needed for people of all age groups, particularly males, who have a higher risk of consuming improperly cooked food. This effort should focus on self-treatment to correct misunderstandings about anti-parasitic drug use. Effective treatment requires proper diagnosis and management, which cannot be achieved through individual empiric treatment practice (self-treatment). Therefore, developing prevention programs in the local communities should focus on these findings to reduce the prevalence of parasitic infections and reduce the risk of cholangiocarcinoma in these communities.

## Figures and Tables

**Table 1:** Socio-demographic data and health behavioral factors related to parasitic infection.

Variables			Total	Parasitic Infection		Non-Parasitic Infection		χ^2^	*p*-Value
				n	(%)	n	(%)		
Age									
	20–39		56	2	(3.6)	54	(96.4)	3.464 ^b^	0.063
	40–59		200	20	(10.0)	180	(90.0)		
	≥60		157	20	(12.7)	137	(87.3)		
Gender									
	Male		186	29	(15.6)	157	(84.4)	11.192 ^a^	0.001 *
	Female		230	13	(5.7)	217	(94.3)		
Education									
	Primary school		289	34	(11.8)	255	(88.2)	1.813 ^b^	0.178
	Secondary school		105	7	(6.7)	98	(93.3)		
	Bachelor degree		12	1	(8.3)	11	(91.7)		
Occupation									
	Agriculturist		144	21	(14.6)	123	(85.4)	4.078 ^a^	0.253
	Labor		93	8	(8.6)	85	(91.4)		
	Merchant		63	4	(6.3)	59	(93.7)		
	Other		84	8	(9.5)	76	(90.5)		
Comorbidity									
	No		226	21	(9.3)	205	(90.7)	0.291 ^a^	0.589
	Yes		193	21	(10.9)	172	(89.1)		
Smoke									
	Yes		50	13	(26.0)	37	(74.0)	18.240 ^a^	<0.001 *
	Ex-smoker		25	5	(20.0)	20	(80.0)		
	No		322	24	(7.5)	298	(92.5)		
Alcohol drinker									
	Yes		93	17	(18.3)	76	(81.7)	10.859 ^a^	0.004 *
	Ex-drinker		42	6	(14.3)	36	(85.7)		
	No		278	19	(6.8)	259	(93.2)		
Number of times a parasitic drug was used in 1 year									
	≥2 time		48	5	(10.4)	43	(89.6)	0.003 ^b^	0.955
	1 time		112	10	(8.9)	102	(91.1)		
	0 time		77	8	(10.4)	69	(89.6)		
Raw food consumption (at least one type of food)									
	Often		221	33	(14.9)	188	(85.1)	6.561 ^a^	0.010 *
	Never or sometimes		145	9	(6.2)	136	(93.8)		
Shoe-wearing outside the house and boot-wearing during farm work									
	Sometimes		154	20	(13.0)	134	(87.0)	1.109 ^a^	0.292
	Always		230	22	(9.6)	208	(90.4)		
Defecation into a toilet at home and on the farm									
	Sometimes		130	20	(15.4)	110	(84.6)	3.664 ^a^	0.056
	Always		248	22	(8.9)	226	(91.1)		

* *p* < 0.05, ^a^ Pearson Chi-square, ^b^ Mantel–Haenzsel test for trend.

**Table 2:** The intestinal parasitic infections among participants in the community.

**Type of Intestinal Parasitic Infection**	**Frequency**	**Percentage**
**Helminth** *Opisthorchis viverrini*	22/419	5.3
*Strongyloides stercoralis*	13/419	3.1
Hookworm	1/419	0.2
*Ascaris lumbricoides*	1/419	0.2
*Echinostoma* spp.	1/419	0.2
Minute intestinal fluke	1/419	0.2
**Protozoan**		
*Giardia lamblia*	1/419	0.2
*Balantidium coli*	1/419	0.2
*Entamoeba coli*	1/419	0.2
Blastocystis hominis *	1/419	0.2

* 1 participant had multiple infections of *Opisthorchis viverrini* and *Blastocystis hominis.*

**Table 3:** The univariable and multivariable logistic regression analysis for parasitic infection in the community.

Variables		OR_crude_	(95%CI)	*p*-Value	OR_adj_	(95%CI)	*p*-Value
Gender							
	Male	3.08	(1.55–6.12)	0.001 *	2.42	(1.00–5.85)	0.049 *
	Female	1			1		
Age							
	≤39	1			1		
	40–59	3	(0.68–13.25)	0.147	4.09	(0.89–18.76)	0.070
	≥60	3.94	(0.89–17.44)	0.071	7.55	(1.60–35.76)	0.011 *
Educational status							
	Primary school	1.47	(0.18–11.72)	0.718			
	Secondary school	0.79	(0.09–6.99)	0.829			
	Bachelor degree	1					
Occupation							
	Agriculturist	1.88	(0.98–3.60)	0.058			
	Others	1					
Smoker							
	Yes	4.36	(2.05–9.30)	<0.001 *	2	(0.74–5.44)	0.174
	Ex-smoker	3.1	(1.07–9.00)	0.037 *	1.52	(0.37–6.21)	0.556
	No	1			1		
Alcohol drinker							
	Yes	3.05	(1.51–6.16)	0.002 *	1.158	(0.65–3.84)	0.318
	Ex-drinker	2.27	(0.85–6.06)	0.101	1.14	(0.31–4.12)	0.843
	No	1			1		
Number of times a parasitic drug was used in 1 year							
	≥2 times	1.00	(0.31–3.27)	0.996			
	1 time	0.85	(0.32–2.25)	0.846			
	0 time	1					
Raw food consumption (at least one type of food)							
	Often	2.65	(1.23–5.73)	0.013*	2.37	(1.03–5.44)	0.041 *
	Never or some times	1			1		
Shoe-wearing outside the house and boot-wearing during farm work							
	Sometimes	1.41	(0.74–2.68)	0.294			
	Always	1					
Defecation into a toilet at home and on the farm							
	Sometimes	1.87	(0.98–3.57)	0.058			
	Always	1					

* *p* < 0.05.

## Data Availability

The datasets used and/or analyzed during the current study are available from the corresponding author upon reasonable request.
